# Impact of Traumatic Dental Injury on the Quality of Life of Brazilian Preschool Children

**DOI:** 10.3390/ijerph10126422

**Published:** 2013-11-28

**Authors:** Maria B. Siqueira, Ramon T. Firmino, Marayza A. Clementino, Carolina C. Martins, Ana F. Granville-Garcia, Saul M. Paiva

**Affiliations:** 1 Department of Dentistry, School of Dentistry, State University of Paraíba, Av. das Baraúnas, 351, Campina Grande, Paraiba 58109753, Brazil; E-Mails: mbldsiqueira@yahoo.com.br (M.B.S.); ramontargino@gmail.com (R.T.F.); marayza84@gmail.com (M.A.C.); 2 Department of Pediatric Dentistry and Orthodontic, School of Dentistry, Federal University of Minas Gerais, Av. Antônio Carlos, 6627 Belo Horizonte, Minas Gerais 31270901, Brazil; E-Mails: carolcm10@hotmail.com (C.C.M.); smpaiva@uol.com.br (S.M.P.)

**Keywords:** quality of life, tooth injuries, child

## Abstract

This study aimed to assess the impact of traumatic dental injury (TDI) on the quality of life of preschoolers and their families. A cross-sectional study was carried out, with a sample of 814 children, aged three to five years old, in Campina Grande, Brazil. Parents/caregivers were asked to complete the Brazilian Early Childhood Oral Health Impact Scale and a questionnaire on socio-demographic data. Oral examinations of the children were performed by three previously calibrated dentists. Bivariate and multiple Poisson regression analyses were performed (α = 5%). The prevalence of negative impact from oral conditions on quality of life was 31.1% among the children and 24.7% among the families. TDI was not associated with a negative impact on quality of life. Parent/caregiver’s assessment of the child’s oral health (PR = 1.210; 95% CI: 1.027–1.426) and history of toothache (PR = 4.997; 95% CI: 2.943–8.493) remained in the final model for the child section, whereas only a history of toothache (PR = 2.791; 95% CI: 1.801–4.325) remained in the final model for the family section. TDI exerted no negative impact on quality of life in the present sample. A history of toothache was the only variable associated with a negative impact on the quality of life of the preschoolers and their families.

## 1. Introduction

Current studies stress the need to consider the functional and psychosocial dimensions of oral health for the implementation and evaluation of public health interventions in dentistry [1]. The assessment of quality of life has become an integral part of the evaluation of health programs, as traditional dental indicators focused on the presence/absence of oral disease do not demonstrate the extent to which such conditions exert an effect on activities of daily living [2,3]. Thus, in addition to clinical measures, information on oral health-related quality of life (OHRQoL) is essential for healthcare policy makers to perform an adequate assessment of oral health needs [4].

Oral diseases and disorders can have an impact on the quality of life of preschool children and their parents, affecting their oral health and wellbeing [5]. The Early Childhood Oral Health Impact Scale (ECOHIS) is a proxy measure for assessing the impact of oral health problems on the quality of life of preschool children and their families in epidemiological surveys [6,7,8]. Parents play an important role in decision-making with regard to their children’s oral health and this assessment tool measures parents’ perceptions on how oral health problems, including symptoms, the disease itself, and its treatment, affect their child’s quality of life [6,9].

With the decline of the prevalence of dental caries, public oral health for children has become more concerned with other oral health issues such as dental trauma injury (TDI) [10], which is the second most prevalent type of dental condition affecting children aged five years or younger [11,12,13,14,15]. TDI can result in pain, loss of function, emotional distress, and can adversely affect the developing occlusion as well as dental esthetics, with a negative impact on the lives of children [16,17]. Few studies have been carried out in Brazil assessing the impact of TDI on the quality of life of preschool children and their families [17,18]. Moreover, there is no consensus with regard to the findings and no population-based studies have been conducted. Thus, little is known regarding the feelings of children with TDI or the emotional and psychological impact of this condition on young children and their families.

The purpose of the present study was to assess the impact of traumatic dental injury on the quality of life of preschool children and their families in northeastern Brazil.

## 2. Methods

### 2.1. Sample Characteristics

A population-based, cross-sectional study was carried out involving 814 male and female children aged three to five years enrolled at preschools (both public and private) in the city of Campina Grande, Brazil. Participants were selected from a total population of 12,705 children in this age group and corresponded to 6.41% of that population. Campina Grande (population: 386,000) is an industrialized city in northeastern Brazil divided into six health districts. The city has considerable cultural, social, and economic disparities, with a mean monthly income of approximately US$110 per capita and a Human Development Index of 0.72 [19].

A two-phase random sampling strategy was used to ensure representativeness. In the first phase, preschools were randomly selected from each health district, and, in the second phase, children were randomly selected from each preschool. Eighteen of the 127 public preschools and fifteen of the 122 private preschools in the city of Campina Grande were randomly selected. The sample size was calculated based on a four percent margin of error, a ninety-five percent confidence level and a fifty percent prevalence rate of impact on child and family OHRQoL. A correction factor of 1.2 was applied to compensate for the design effect [20]. The minimum sample size was estimated at 720 schoolchildren, to which a further twenty percent was added to compensate for possible losses, giving a total sample of 864 schoolchildren, who were randomly selected from the previously selected schools for participation in the study.

### 2.2. Eligibility Criteria

The following were the inclusion criteria: age three to five years old; enrollment in a preschool or daycare center; absence of systemic disease according parent/caregiver’s information; being accompanied by a Brazilian Portuguese language-speaking caregiver; agreement to participate through a signed statement of informed consent; and the return of completed questionnaires. The exclusion criterion was having four missing maxillary incisors due to caries or physiological exfoliation, which could compromise the clinical diagnosis of TDI.

### 2.3. Training and Calibration Exercise

The calibration exercise consisted of two steps, both theoretical and clinical. The theoretical step involved a discussion of the criteria for the diagnosis of TDI and malocclusion, the administration of the ICDAS II, and an analysis of photographs. A specialist in pediatric dentistry (the gold standard in this theoretical framework) coordinated this step, instructing three general dentists on how to perform the examination. Cases of disagreement were discussed with the group of dentists who participated in the exams prior to the clinical step. The clinical step was performed at a randomly selected preschool that was not part of the main sample. Each dentist examined 50 previously selected children between three to five years of age. Inter-examiner agreement was tested by comparing each examiner with the gold standard (K = 0.85 to 0.90). A seven-day interval was respected between clinical examinations for the determination of intra-examiner agreement (K = 0.85 to 0.90). Data analysis involved Cohen’s Kappa coefficient occurred on a tooth-by-tooth basis. As Kappa coefficients were very good [21], the examiners were considered capable of performing the epidemiological study.

### 2.4. Pilot Study

A pilot study was performed to test the methodology and comprehension of the questionnaires. The children in the pilot study (*n* = 40) were not included in the main sample. As there were no misunderstandings regarding the questionnaires or the methodology, no changes needed to be made to the data collection process.

### 2.5. Non-Clinical Data Collection

The Early Childhood Oral Health Impact Scale (ECOHIS) and questionnaires addressing socio-demographic and health data of children questionnaires were filled out by parents/caregivers. The ECOHIS assesses parents/caregivers’ perceptions regarding the negative impact of oral health problems on the quality of life of preschool children and their families. This scale is divided into two sections; child impact and family impact, with six domains and thirteen items. The domains in the Child Impact Section are symptoms (one item), function (four items), psychological (two items) and self-image/social interaction (two items). The domains in the Family Impact Section are distress (two items) and family function (two items). Each item has six response options: 0 = never, 1 = hardly ever, 2 = occasionally, 3 = often, 4 = very often and 5 = don’t know. Item scores are summed for each section (“don’t know” responses are not counted). The total score ranges from 0 to 36 in the Child Impact Section and 0 to 16 in the Family Impact Section, with higher scores indicating greater impacts and/or more problems. The Brazilian version of the ECOHIS has been validated in Brazilian Portuguese and used in previous studies [8,18,22]. There were two dependent variables: the impact on children’s OHRQL and impact on the family’s OHRQL. The presence of impact on children’s QoL was considered when at least one answer “hardly ever” was giver in any of four domains (symptom, functional, psychological, self-image/social). The absence of impact was when all answers were given as “never”. The presence of impact of a family’s QoL was considered when at least one answer of “hardly ever” was given in any of two domains (distress and family function). An absence of impact on family’ QoL was when all domains were answered “never” [6,17,23,24].

Socio-demographic data: Parent/caregiver’s age and years of schooling, number of people in the home; type of school; monthly household income (categorized based on the minimum salary in Brazil = US$312.50)

Child health data: Parent/caregiver’s assessment of child’s general and oral health; history of toothache; history of dental visits; and history of trauma (parents/caregivers of children with a normative diagnosis of trauma).

### 2.6. Clinical Data Collection

The clinical examination was performed at the preschool after the return of the questionnaires and after having received signed informed consent. The examinations were performed by three dentists who had undergone the calibration exercise. Prior to the clinical exam, the children brushed their teeth under the examiner’s supervision. For such, each child received a kit containing a toothbrush, toothpaste, and dental floss to remove bacterial biofilm from the dental surfaces and facilitate the diagnosis. Lip seal was evaluated prior to the intraoral examination without the subject aware that he or she was being observed and was determined adequate when contact between the lips occurred in the resting position with the teeth in occlusion [25].

Oral examinations were performed in the knee-to-knee position with the aid of a portable lamp attached to the examiner’s head (Petzl Zoom head lamp, Petzl America, Clearfield, UT, USA). The dentists used individual cross-infection protection equipment as well as packaged, sterilized mouth mirrors (PRISMA^®^, Sao Paulo, SP, Brazil), Williams’ probes (WHO-621, Trinity^®^, Campo Mourão, PA, Brazil) and dental gauze. To measure overjet, the examiner placed a Williams’ periodontal probe on the incisal surface of the maxillary central incisors parallel to the occlusal plane to determine the horizontal relation of the incisors. This measurement was taken with the teeth in centric occlusion. Overjet was dichotomized as (i) 2 mm or less (normal overjet); and (ii) greater than 2 mm (accentuated overjet) [26]. Open bite was recorded when the anterior teeth were not in contact with the posterior teeth in occlusion [27]. The classification proposed by Andreasen *et al*. [28] was used for the clinical diagnosis of TDI: enamel fracture, enamel and dentin fracture, complicated crown fracture, extrusive luxation, lateral luxation, intrusive luxation, and avulsion. A visual assessment of tooth discoloration was also performed. Dental caries was diagnosed using the International Caries Detection and Assessment System (ICDAS II) [29]; the first visual change in enamel was considered as caries. Children with at least one of the following conditions were classified as having malocclusion: overbite [26], accentuated overjet [26,30], and posterior crossbite recognized first by Foster and Hamilton [31]. Following the examination, fluoride varnish was applied for all children and those with carious lesions or other dental needs were sent for treatment.

### 2.7. Statistical Analysis

Simple descriptive statistics were performed to characterize the sample and demonstrate the distribution of ECOHIS items. Bivariate analysis was performed using the chi-square test to determine associations between TDI and negative impacts on the ECOHIS items. Bivariate Poisson regression analysis with robust variance was employed to determine associations between the independent variables and negative impact on the quality of life on the children and their families (*p* < 0.05). Multivariate Poisson regression models were constructed after controlling for the confounding effects of dental caries and malocclusion. Forward stepwise multivariate Poisson regression models were constructed with variables having achieved a *p*-value < 0.20 in the bivariate analysis as well as variables considered epidemiological determinants, after controlling for the confounding effects of dental caries and malocclusion. This analysis was performed with two dependent variables at a time; Impact on Quality of Life of the Child and Impact on Quality of Life of the Family. Data organization and statistical analyses were carried out using the Statistical Package for Social Sciences (SPSS for Windows, version 18.0, SPSS Inc., Chicago, IL, USA).

### 2.8. Ethical Considerations

The present study received approval from the Human Research Ethics Committee of the State University of Paraíba (Campina Grande, Brazil) under process number 00460133000-11 in compliance with Resolution 196/96 of the Brazilian National Health Council. 

**Figure 1:**
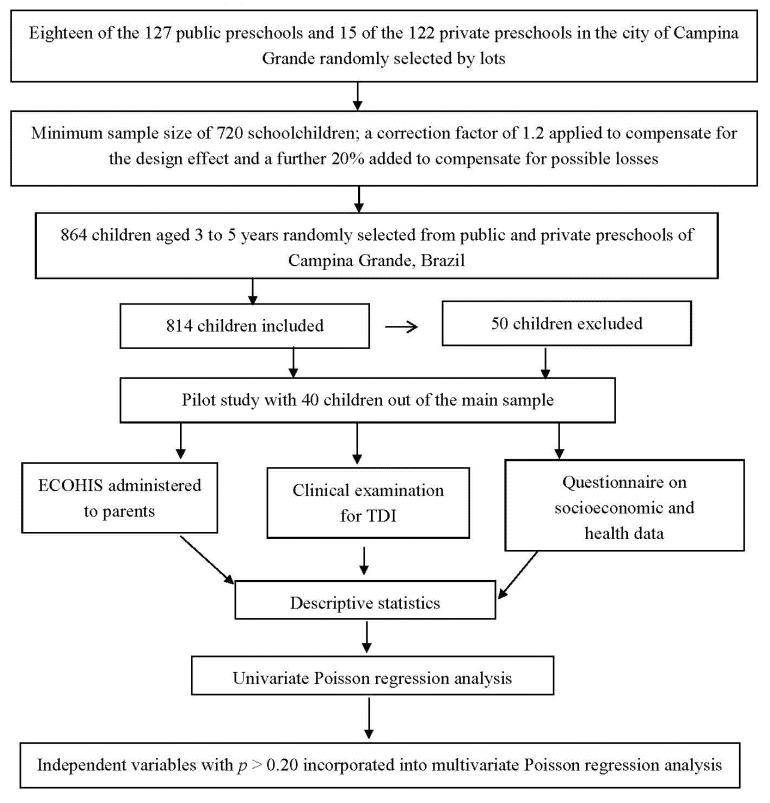
Flowchart of study.

The flow chart of the study (Figure 1):

## 3. Results

Among the 864 selected, 814 participated in the present study, corresponding to 94.22% of the total determined by the sample calculation process. The exclusion of 50 children was due to a lack of participation on the part of the child for medical reasons (2), incomplete questionnaires (15), absence from preschool/daycare center on the days scheduled for the clinical exams (15), and a lack of cooperation during the clinical exam (18). Table 1 displays the socio-demographic and clinical data of the sample. The prevalence of TDI was 34.6%. The upper central incisors were the most affected (88.4%), followed by lateral central incisors (8.9%). Only 10 affected teeth (2.7%) were lower incisors. Enamel fracture was the most common type of TDI (17.0%), followed by tooth discoloration (11.2%). Most children had only one tooth affected (21.7%).

**Table 1:** Frequency distribution of preschool children according to independent variables.

Variable	Frequency	
	N	%
**• Gender of child**		
Female	392	48.2
Male	422	51.8
**• Number of residents in home**		
<to 6 residents	674	84.6
6 or more residents	123	15.4
**• Household income**		
>3 times the minimum wage	144	18.5
≤3 times the minimum wage	634	81.5
**• Parent/caregiver’s schooling**		
>8 years of study	437	54.0
≤8 years of study	373	46.0
**• Parent/caregiver’s assessment of child’s oral health**		
Good	759	93.4
Poor	54	6.6
**• Parent/caregiver’s assessment of child’s general health**		
Good	801	99.0
Poor	8	1.0
**• TDI**		
Yes	281	34.6
No	533	65.4
**• Type of TDI**		
None	533	65.4
Tooth discoloration	91	11.2
Enamel fracture	138	17.0
Enamel + dentin fracture	39	4.8
Luxation	9	1.1
Avulsion	4	0.5
**• Number of teeth affected by TDI**		
2 or more teeth	104	12.8
1 tooth	177	21.7
None	533	65.5
**• History of toothache**		
Yes	71	36.6
No	123	63.4
**• History of dental visits**		
Yes	196	24.2
No	614	75.8
**• Type of preschool**		
Public	438	53.8
Private	376	46.2

The prevalence of negative impact on quality of life was 31.1% and 24.7% among children and their families, respectively. The items with the greatest frequency of the Child Impact Section of the ECOHIS were “related to pain” (21.6%), “had difficulty eating some foods” (12.5%), and “had difficulty drinking hot or cold beverages” (12.4%). The items with the greatest frequency of the Family Impact Section were “felt guilty” (17.3%) and “been upset” (14%) (Table 2).

**Table 2:** Prevalence of impact of oral health on quality of life and ECOHIS scores among preschool children.

ECOHIS				
Domains, Items	SCORE Mean ± DP	Minimum–Maximum	n (%) Don’t Know	n (%) Prevalence of impact
**• Child Impact**	2.21 ± 4.18	0–31	-	31.1%
Related to pain	0.58 ± 0.979	0–4	14 (1.7%)	176 (21.6%)
Had difficulty drinking hot or cold beverages	0.35 ± 0.816	0–4	10 (1.2%)	101 (12.4%)
Had difficulty eating some foods	0.35 ± 0.830	0–4	11 (1.4%)	103 (12.5%)
Had difficulty pronouncing words	0.21 ± 0.707	0–4	25 (3.1%)	59 (7.2%)
Missed preschool, daycare or school	0.12 ± 0.466	0–3	-	29 (3.6%)
Had trouble sleeping	0.17 ± 0.596	0–4	8 (1.0%)	45 (5.5%)
Been irritable or frustrated	0.29 ± 0.745	0–4	8 (1.0%)	85 (10.4%)
Avoided smiling or laughing	0.08 ± 0.395	0–4	10 (1.2%)	18 (2.2%)
Avoided talking	0.08 ± 0.00	0–4	9 (1.1%)	19 (2.3%)
**• Family Impact**	1.13 ± 2.167	0–14		24.7%
Been upset	0.39 ± 0.902	0–4	11 (1.4%)	114 (14.0%)
Felt guilty	0.46 ± 0.938	0–4	11 (1.4%)	141 (17.3%)
Taken time off work	0.16 ± 0.564	0–4	8 (1.0%)	47 (5.8%)
Financial impact	0.14 ± 0.560	0–4	9 (1.1%)	41 (5.0%)

No significant associations were found between the presence of TDI and isolated items on the ECOHIS (Table 3).

**Table 3:** Frequency distribution of preschool children with or without TDI according to each ECOHIS item.

ECOHIS	TDI						
	Yes		No		Total		*p*-value
Domains, Items	N	%	N	%	N	%	
**• Child Impact**							
No impact	185	(33.0)	376	(67.0)	561	(68.9)	0.45
Impact	92	(36.4)	161	(63.6)	253	(31.1)	
***• Symptoms Domain***							
** Related to Pain**							
No impact	218	(34.2)	420	(65.8)	638	(78.4)	0.873
Impact	59	(33.5)	117	(66.5)	176	(21.6)	
***• Function Domain***							
** Had difficulty drinking hot or cold beverages**							
No impact	236	(33.6)	467	(66.4)	703	(87.4)	0.543
Impact	37	(36.6)	64	(63.4)	101	(12.6)	
** Had difficulty eating some food**							
No impact	235	(33.5)	466	(66.5)	462	(81.9)	0.724
Impact	36	(35.3)	66	(64.7)	102	(18.1)	
** Had difficulty pronouncing words**							
No impact	239	(32.7)	491	(67.3)	730	(92.5)	0.213
Impact	24	(40.7)	35	(59.3)	59	(7.5)	
** Missing preschool**							
No impact	266	(33.9)	519	(66.1)	785	(96.4)	0.652
Impact	11	(37.9)	18	(62.1)	29	(3.6)	
***• Psychological Domain***							
** Had trouble sleeping**							
No impact	257	(33.8)	504	(66.2)	761	(94.4)	0.952
Impact	15	(33.3)	30	(66.7)	45	(5.6)	
** Been irritable or frustrated**							
No impact	244	(33.8)	477	(66.2)	721	(95.7)	0.959
Impact	29	(34.1)	56	(65.9)	32	(4.3)	
***• Self-image/Social Interaction Domain***							
** Avoided smiling or laughing**							
No impact	264	(63.6)	522	(66.4)	786	(97.8)	0.336
Impact	8	(44.8)	10	(55.6)	18	(2.2)	
** Avoided talked**							
No impact	265	(33.7)	521	(66.3)	786	(97.7)	0.445
Impact	8	(42.1)	11	(57.9)	19	(2.3)	
** Family Impact**							
No impact	205	(33.7)	403	(66.3)	608	(75.1)	0.874
Impact	69	(34.3)	132	(65.7)	201	(24.9)	
***• Distress Domain***							
** Been upset**							
No impact	229	(33.2)	460	(66.8)	689	(85.8)	0.349
Impact	43	(37.7)	71	(62.3)	114	(14.2)	
** Felt guilty**							
No impact	227	(34.3)	435	(65.7)	662	(82.4)	0.705
Impact	46	(32.6)	95	(67.4)	141	(17.6)	
***• Family Function Domain***							
** Taken time off work**							
No impact	256	(33.7)	503	(66.3)	759	(94.2)	0.521
Impact	18	(38.3)	29	(61.7)	47	(5.8)	
** Financial Impact**							
No impact	260	(34.0)	504	(66.0)	764	(94.9)	0.759
Impact	13	(31.7)	28	(68.3)	41	(5.1)	

In the bivariate analysis, the following variables were associated with the prevalence of impact on the quality of life of the child: lower level of mother’s schooling; lower household income; greater number of residents in the home; attending a private preschool/daycare center; poorer evaluation of parents/caregivers regarding child’s general and oral health; presence of TDI; tooth discoloration; luxation; history of visiting the dentist; and history of toothache. However, only the parent/caregiver’s evaluation regarding the child’s oral health and a history of toothache remained in the final Poisson multiple regression model (Table 4).

**Table 4:** Frequency distribution and Poisson regression analysis according to independent variables and impact on quality of life (QoL) of preschool children.

Variable	Impact on child’s QoL		Bivariate		Multivariate	
	Present	Absent	Unadjusted prevalence ratio		Adjusted prevalence ratio	
	n (%)	n (%)	*p*-value	(95% CI)	*p*-value	(95% CI)
**• Gender of child**						
Male	131 (31.0)	291 (69.0)		1.00	-	-
Female	122 (31.1)	270 (68.9)	0.980	1.003 (0.817–1.230)	-	-
**• Mother’s schooling**						
>8 years of study	113 (25.9)	324 (74.1)		1.00	-	-
≤8 years of study	139 (37.3)	234 (62.7)	0.001	1.441 (1.173–1.771)	-	-
**• Monthly household income**						
>3 times the minimum wage	29 (20.1)	115 (79.9)		1.00	-	-
≤3 times the minimum wage	219 (34.5)	415 (65.5)	0.020	1.715 (1.218–2.416)	-	-
**• N° of residents in home**						
<6	199 (29.5)	475 (70.5)		1.00	-	-
≥6	50 (40.7)	73 (59.3)	0.010	1.377 (1.079–1.756)	-	-
**• Type of school**						
Public	153 (34.9)	285 (65.1)		1.00	-	-
Private	100 (26.6)	276 (73.4)	0.011	1.313 (1.064–1.622)	-	-
**• Caregiver’s perception of child’s general health**						
Good	247 (30.8)	554 (69.2)		1.00		-
Poor	6 (75.0)	2 (25.0)	0.001	2.432 (1.609–3.677)	-	-
**• Caregiver’s perception of child’s oral health**						
Good	205 (27.0)	554 (73.0)		1.00		1.00
Poor	48 (88.9)	6 (11.1)	0.001	2.432 (1.609–3.677)	0.23	1.210 (1.027-1.426)
**• TDI**						
Yes	95 (33.8)	186 (66.2)		1.00	-	-
No	158 (29.6)	357 (70.4)	0.342	1.108 (0.897–1.368)	-	-
**• Type of trauma**						
Avulsion/Luxation	6 (46.2)	7 (53.8)	0.139	1.572 (0.864–2.861)	-	-
Discoloration	37 (40.7)	54 (59.3)	0.020	1385 (1.052–1.822)	-	-
Enamel + dentin fracture	13 (33.3)	26 (66.7)	0.588	1.135 (0.717–1.797)	-	-
Enamel fracture or no trauma	197 (29.4)	474 (70.6)		1.00	-	-
**• Number of teeth with trauma**						
None	158 (29.6)	375 (70.4)		1.00	-	-
One	52 (29.4)	125 (70.6)	0.947	0.991 (0.762–1.289)	-	-
Two or more	43 (41.3)	61 (58.7)	0.013	1.395 (1.072–1.816)	-	-
**• Dental caries**						
No	45 (16.1)	235 (83.9)	<0.001	2.424 (1.817–3.232)	-	-
Yes	208 (39.0)	326 (61.0)		1.00	-	-
**• Malocclusion**						
No	80 (28.6)	200 (71.4)		1.00	-	-
Yes	173 (32.4)	361 (67.6)	0.267	1.134 (0.908–1.416)	-	-
**• History of toothache**						
No	15 (12.2)	108 (87.8)	-	1.00	-	1.00
Yes	62 (87.3)	9 (12.7)	0.001	7.161 (4.420–11.600)	0.001	4.997 (2.943-8.483)
**• History of visits to dentist**						
No	173 (28.2)	441 (71.8)		1.00	-	-
Yes	78 (39.8)	118 (60.2)	0.002	1.412 (1.41–1.749)	-	-

In the bivariate analysis, the following variables were associated the prevalence of impact on the quality of life of the family: lower level of the mother’s schooling; greater number of residents in the home; poorer evaluation of parents/caregivers regarding child’s general and oral health; tooth discoloration; luxation; history of visiting the dentist; and history of toothache. However, only history of toothache remained in the final Poisson multiple regression model (Table 5).

**Table 5:** Frequency distribution and Poisson regression analysis according to independent variables and impact on quality of life (QoL) of family of preschoolers.

Variable	Impact on family’s QoL			Bivariate				Multivariate		
	Present		Absent	Unadjusted prevalence ratio				Adjusted prevalence ratio		
	n (%)		n (%)	*p*-value			(95% CI)	*p*-value		(95% CI)
**• Gender of child**										
Male	94 (24.2)		294 (75.8)				1.00	-		-
Female	107 (25.4)		314 (74.6)	0.696			1.049 (0.825–1.334)	-		-
**• Mother’s schooling**										
>8 years of study	97 (22.3)		338 (77.7)				1.00	-		-
≤8 years of study	103 (27.8)		267 (72,2)	0,070			1.248 (0.982–1.587)	-		-
**• Monthly household income**										
>3 times the minimum wage	31 (21.5)		113 (78.5)				1.00	-		-
≤3 times the minimum wage	165 (26.2)		464 (73.8)	0.252			1.219 (0.869–1.709)	-		-
**• N° of residents in home**										
<6	160 (23.9)		510 (76.1)				1.00	-		-
≥6	38 (30.9)		85 (69.1)	0.089			1.294 (0.961–1.741)	-		-
**• Type of school**										
Public	85 (22.8)		287 (77.2)				1.00	-		-
Private	116 (26.5)		321 (73.5)	0.227			1.162 (0.911–1.482)	-		-
**• Caregiver’s perception of child’s general health**										
Good	194 (24.4)		602 (75.6)				1.00	-		-
Poor	5 (62.5)		3 (37.5)	0.001			2.564 (1.479–4.447)	-		-
**• Caregiver’s perception of child’s oral health**										
Good	161 (21.4)		593 (78.6)				1.00	-		-
Poor	40 (74.1)		14 (25.9)	0.001			3.469 (2.815–4.275)	-		-
**• TDI**										
Yes	131 (24.7)		400 (75.3)				1.00	-		-
No	70 (25.2)		208 (74.8)	0.874			1.121 (0.793–1.313)			-
**• Type of trauma**										
Avulsion/Luxation		5 (38.5)	8 (61.5)		0.185	1.606 (0.797–3.236)			-	-
Discoloration		28 (31.5)	61 (68.5)		0.111	1.313 (0.939–1.836)			-	-
Enamel + dentin fracture		8 (20.5)	31 (79.5)		0.631	0.856 (0.455–1.612)			-	-
Enamel fracture or no trauma		160 (24.0)	508 (76.0)			1.00			-	-
**• Number of teeth with trauma**										
None		131 (24.7)	400 (75.3)			1.00			-	-
One		40 (23.0)	134 (77.0)		0.655	0.932 (0.683–1.270)			-	-
Two or more		30 (28.8)	74 (71.2)		0.362	1.169 (0.835–1.637)			-	-
**• Dental caries**										
No		34 (12.3)	243 (87.7)		<0.001	2.557 (1.822–3.589)			0.022	2.305 (1.130–4.702)
Yes		167 (31.4)	365 (68.6)			1.00			-	1.00
**• Malocclusion**										
No		61 (22.0)	216 (78.0)			1.00			-	-
Yes		140 (26.3)	392 (73.7)		0.185	1.195 (0.918–1.555)			-	-
**• History of toothache**										
No		22 (18.0)	100 (82.0)		-	1.00				1.00
Yes		48 (67.6)	23 (32.4)		0.001	3.749 (2.485–5.656)			0.001	2.791 (1.801–4.325)
**• History of visits to dentist**										
No		129 (21.1)	481 (78.9)			1.00			-	-
Yes		71 (36.4)	124 (63.6)		0.001	1.772 (1.354–2.190)			-	-

## 4. Discussion

OHRQoL assessment tools have been employed with increasing frequency in oral health surveys [32]. Dental disease and treatment experience can negatively affect the OHRQL of preschool children and their parents/caregivers [6]. The ECOHIS is a proxy measure of children’s OHRQoL [8] for which parents/caregivers are the secondary respondents, as it is believed that very young children do not have sufficient cognitive skills to evaluate their own quality of life [6,24]. This method has been validated in the existing literature [6,8,22,33].

The prevalence of negative impact on quality of life of the child was 31.1%, which is lower than the figure reported in previous Brazilian studies (49% to 69.3%) [5,17,18]. The divergences may be explained by differences in the sample profile and methods employed. In studies with a prevalence rate as high as 69.3% [5,17], the samples were not population-based and were made up of children treated at healthcare services, which may have influenced the responses. In the study with a prevalence rate of 49% [18], the sample was randomly selected but not representative. Moreover, in all three studies, responses of “hardly ever” were recorded as the presence of impact, which likely increased the prevalence of negative impact on quality of life. In the present study, the decision was made to consider the presence of impact only when answers of “occasionally”, “often”, and “very often” were recorded [6]. This point merits attention, as previous investigations have not defined the cutoff point for the presence/absence of a negative impact on quality of life using the ECOHIS. Some studies use the mean or median ECOHIS score to determine the negative impact and associations with the variables analyzed [7,17,34,35]. Other studies use a qualitative categorization (yes/no), but with adaptation to the questionnaire (combination of items) [36] or cutoff points that differ from that used in the present study [17,18,35], which hinders the comparison of the results.

In the analysis, the most frequent responses on the Child Impact Section of the ECOHIS included “related to pain”, “had difficulty drinking hot or cold beverages”, and “had difficulty eating some foods”. This is similar to findings reported in other Brazilian studies [8,18,35] as well as a study carried out in China [7]. The symptom and function domains seem to be more perceptible to family members than the domains concerning psychology and self-image. On the other hand, studies report that children in this age group do not have sufficient psychological maturity or self-image to establish comparisons with other children; thus, the consequences of oral conditions may be minimized with regard to these aspects [5,37]. The structure of a child’s self-concept is age dependent as a result of continuous cognitive, emotional, social and language development [38]. Child developmental psychology explains that the age of six years is marked by the onset of abstract thinking and self-awareness [37]. Indeed, emotional status and aesthetics are considered to be factors associated with a negative impact on quality of life among older age groups. A Brazilian study involving adolescents aged 11 to 14 years using the Child Perceptions Questionnaire (CPQ11-14) found that adolescents with aesthetic abnormalities experienced a negative impact on social wellbeing, mainly with regard to avoid smiling or laughing and being concerned about what other people may think or say [3]. A similar finding is reported in a study carried out in Great Britain involving adolescents aged 16 and 17 years using the Oral Health Impact Profile (OHIP-14), in which aesthetic abnormalities constituted a major self-reported cause of impact on daily performances [39]. However, the comparison of quality of life studies involving different age groups is hampered by the methods employed. While studies involving adolescents employ self-reports of impact, investigations involving children aged five years or younger rely on proxy reports from parents/guardians which may exert an influence on the results [18]. Indeed, there are reports in the literature regarding the lack of importance some parents/caregivers place on the primary dentition [13,40], which also may have contributed to the findings.

The prevalence of negative impact on the quality of life of the family was 24.7%. Once again, this is lower than the figure reported in previous Brazilian studies (30.7% to 35%) [5,17,18]. The reasons for these divergences are likely the same as those reported with regard to the Child Impact Section. Another cross-sectional study carried out in Brazil specifically analyzing the impact of oral health conditions among children on families reports a negative impact of 87.3% [35]. However, the study cited was conducted at a healthcare service involving children with a high percentage of accumulated treatment needs, which likely influenced the results. In contrast, the present study involved a population-based sample at preschools.

In analyzing each item in the Family Health Section, “felt guilty” and “been upset” were the most cited, which is in line with a tendency observed in previous studies [5,7,18]. In an investigation conducted in the United States, the most frequent responses were “taken time off work” and “felt guilty” [6]. The greater participation of women in the job market in recent decades, which has led to delegating the care of children’s health to third parties [41], and the lack of knowledge regarding the need to visit the dentist in the early years of a child’s life [42] likely affect the feelings of parents/caregivers in terms of the negative impact on the quality of life of the family. A number of authors report that mothers are aware of their responsibilities with regard to providing oral health care and express feelings of guilt, concern, anger, and despair associated with the adverse oral conditions of their children [43,44].

The prevalence of TDI was 34.6% and the characterization of trauma was in agreement with that described in the literature [12,13,45]. It should be pointed out that this prevalence rate may have been underestimated due to the self-correction of some types of past trauma, which were therefore not diagnosed at the time of the exam. This constitutes one of the limitations of the cross-sectional study design. TDI was not significantly associated with the domains of the ECOHIS. Most cases of trauma were mild fractures, which may have influenced the results. Likewise, previous studies have found no association between TDI and the different domains of the ECOHIS [18,35] and also report mild trauma in most cases. However, one cannot discard the possibility of memory bias [46], which is a limitation of cross-sectional studies; many parents/caregivers may have forgotten the details related to the time at which the traumatic event occurred. Another Brazilian study found an association between severe TDI (complicated crown fracture) in the primary dentition and a negative impact on quality of life [17]. The study cited was carried out at a healthcare service and, according to the authors, the association between TDI and negative impact on quality of life was likely due to symptoms frequently related to complicated TDI, such as pain, irritation, difficulty eating some foods, trouble sleeping, and difficulty drinking hot or cold beverages.

The Poisson multivariate analysis controlled for the presence of dental caries and malocclusion. Thus only a parent/caregiver’s evaluation of the child’s oral health and a history of toothache remained associated with a negative impact on quality of life in the Child Section of the ECOHIS. For public health purposes, the evaluations of parents/caregivers regarding their children’s oral health should be incorporated into population-based surveys aimed at assessing the need for dental care among children of preschool age [9]. Children’s self perceptions of their oral own health status was reported to be a strong predictor of negative impact on quality of life in a Brazilian study employing the CPQ11-14 [1]. A study carried out in the United States with children aged four to 12 years of age which used the Michigan Oral Health-Related Quality of Life Scale reports that poor oral health may prevent children from expressing positive emotions, which can impact their social interactions and the way they feel about themselves [47]. A validation study for the Oral Impacts on Daily Performance scale for children (Child-OIDP) carried out in Saudi Arabia involving 12-year-olds found that the perception of oral health plays an important role in the determination of negative impact on quality of life [4]. Regarding a history of toothache, previous Brazilian studies point to a strong association between this variable and the search for dental treatment in preschool children [43,48]. Toothache was the most frequently associated cause of nearly all impacts in both private and public school attendees in the Saudi Arabian study using the Child-OIDP on 12-year-olds [4]. In India, a study reports an 85% prevalence rate of negative impact on activities of daily living due to toothache among 12-year-olds [49]. In a study with a similar methodology as that employed in the present investigation [18], the variables associated with a negative impact on quality of life in the Child Impact Section were the presence of caries, social indicators, and a history of trauma reported by parents/caregivers. However, the normative diagnosis of TDI was not associated with a negative impact on quality of life and the authors did not investigate a history of toothache or oral health perceptions. In the present study, the normative diagnosis of trauma was considered rather than the perception of trauma reported by parents/caregivers, which likely influenced the findings and could be considered a limitation of this study, as the impact on quality of life occurs beginning with the moment at which the trauma is perceived by the parent/caregiver and not when diagnosed by the dentist. Nonetheless, other authors have employed this same methodology [5,17,35].

On the Family Impact Section, dental caries and history of toothache were the variables associated with a negative impact on quality of life in the multivariate model, further demonstrating the value of this factor. The disregard for the deciduous dentition as well the lack of knowledge on the need to visit the dentist in the early years of a child’s life and the etiological factors of adverse oral conditions may have contributed to this finding [43,48]. Indeed, a number of Brazilian studies report low rates of seeking dental care for children aged five years or younger [24,42,48]. Thus, greater investment is needed to raise awareness among parents/caregivers regarding the importance of the primary dentition and routine dental care for preschool children. The greater frequency of responses of “felt guilty” and “been upset” may reflect this result and may be related to untreated caries rather than TDI *per se*, as most cases of dental trauma were mild and not associated with a negative impact on quality of life.

Although social indicators have been reported to be predisposing factors for impact on the quality of life of preschoolers [5,7,8,35], no such association was found in the present study. The studies cited were carried out in different regions/countries, which may have influenced the results. Predictors of a negative impact on quality of life may vary across populations and these aspects should be taken into account in decision-making processes regarding the allocation of resources for healthcare programs [50].

## 5. Conclusions

The present findings reveal that TDI exerted no negative impact on the quality of life of preschoolers and their families. As this investigation was a population-based study, the findings may be extrapolated to the population. The evaluation of parents/caregivers regarding the oral health of their children and a history of toothache were associated with a negative impact on quality of life of the Children Impact Section. Dental caries and history of toothache were associated with a negative impact on the Family Impact Section.
